# Concurrent Use of Tobacco and Cannabis and Internalizing and Externalizing Problems in US Youths

**DOI:** 10.1001/jamanetworkopen.2024.19976

**Published:** 2024-07-03

**Authors:** Vuong V. Do, Pamela M. Ling, Benjamin W. Chaffee, Nhung Nguyen

**Affiliations:** 1 Center for Tobacco Control Research and Education, Cardiovascular Research Institute, University of California, San Francisco; 2 Division of General Internal Medicine, Department of Medicine, University of California, San Francisco; 3 School of Dentistry, University of California, San Francisco

## Abstract

**Importance:**

Little is known about whether concurrent use of tobacco and cannabis is associated with higher or lower levels of mental health problems than use of either substance alone among youths.

**Objective:**

To examine the association between concurrent use of tobacco and cannabis and mental health problems in a national sample of US youths.

**Design, Setting, and Participants:**

This cross-sectional study analyzed publicly available wave 6 data within the Population Assessment of Tobacco and Health (PATH) Study, a nationally representative cohort study of US youths aged 14 to 17 years, collected from March to November 2021. This analysis used wave 6 single-wave weights to obtain statistically valid estimates for cross-sectional analyses generalizable to the wave 4 cohort sample. Data were analyzed from November 15, 2023, to April 20, 2024.

**Exposure:**

Past 30-day use of any tobacco and cannabis products was self-reported and categorized into 4 exclusive patterns: concurrent, tobacco only, cannabis only, and nonuse.

**Main Outcomes and Measures:**

Self-reported past-year internalizing and externalizing mental health problems were measured using the modified version of the Global Appraisal of Individual Needs–Short Screener and separately categorized into 3 levels: low (0-1 symptoms), moderate (2-3 symptoms), and high (≥4 symptoms).

**Results:**

A total of 5585 youths were included in the study, with a weighted proportion of 51.3% being male and 72.5% aged 15 to 17 years. In terms of race and ethnicity, 1606 youths (25.7%) were Hispanic, 626 (12.7%) were non-Hispanic Black, 2481 (50.5%) were non-Hispanic White, and 555 (11.0%) were non-Hispanic other. The prevalence of concurrent use (3.4% [95% CI, 2.9%-4.0%]) was comparable to tobacco-only use (3.9% [95% CI, 3.2%-4.6%]), but greater than cannabis-only use (2.5% [95% CI, 2.1%-2.9%]). High levels of internalizing and externalizing problems were most common for the concurrent use group (internalizing: 47.4% [95% CI, 39.2%-55.9%]; externalizing: 61.6% [95% CI, 54.1%-68.7%]), followed by the cannabis-only use group (internalizing: 44.8% [95% CI, 35.7%-54.1%]; externalizing: 48.5% [95% CI, 39.1%-57.9%]), the tobacco-only use group (internalizing: 41.4% [95% CI, 33.7%-49.5%]; externalizing: 46.3% [95% CI, 38.3%-54.5%]), and the nonuse group (internalizing: 22.4% [95% CI, 21.1%-23.8%]; externalizing: 30.4% [95% CI, 28.9%-31.9%]). After controlling for covariates in ordinal logistic regression models, concurrent use of tobacco and cannabis was associated with greater odds of reporting higher levels of externalizing problems compared with tobacco-only use (adjusted odds ratio [AOR], 1.83 [95% CI, 1.15-2.91]) and cannabis-only use (AOR, 1.85 [95% CI, 1.11-3.06]). However, there were no statistically significant differences in the odds of internalizing problems between concurrent use and use of tobacco or cannabis alone.

**Conclusions and Relevance:**

In this cross-sectional study of the PATH Study wave 6 youth data, concurrent use of tobacco and cannabis was linked to higher levels of externalizing mental health problems than use of single substances, indicating a potential need to combine mental health support with tobacco and cannabis cessation interventions for youths.

## Key Points

**Question:**

Is concurrent use of tobacco and cannabis among youths associated with higher or lower levels of mental health problems than the use of either substance alone?

**Findings:**

In this cross-sectional study of a national sample of 5585 youths aged 14 to 17 years, concurrent use was associated with almost twice the odds of reporting higher levels of externalizing problems compared with tobacco or cannabis use exclusively, but this association did not hold for internalizing problems.

**Meaning:**

These findings suggest that integrated treatment of mental health and tobacco and cannabis use may be beneficial to address these comorbidities among youths.

## Teaser

This cross-sectional study examines the prevalence of tobacco and cannabis use patterns and their associations with internalizing and externalizing mental health problems among participants in the Population Assessment of Tobacco and Health Study aged 14 to 17 years.

## Introduction

Tobacco and cannabis are two of the most commonly used substances among US youths.^1,2^ According to data from the National Survey on Drug Use and Health (NSDUH),^2^ 7.3% of youths aged 12 to 17 years (or 1.9 million people) reported past 30-day tobacco use, and 6.4% (or 1.6 million youths) reported past 30-day cannabis use in 2022. In addition, another study using the 2014 NSDUH data^3^ showed that the prevalence among youths (5.4%) who reported concurrently using both tobacco and cannabis within the past month was greater than the exclusive use of tobacco (3.9%) or cannabis (2.2%). However, these data from the NSDUH do not capture the use of newer products (eg, e-cigarettes and vaporized cannabis) among youths. Given the current context of increasing use of vaporized products for tobacco and cannabis among youths,^4^ more recent data are critical to inform the surveillance of concurrent use of tobacco and cannabis in this age group.

Depression, anxiety, and behavioral disorders are among the leading causes of illness and disability among youth.^5^ In recent years, there have been reports of a youth mental health crisis.^6,7^ Studies among youths have examined the associations between mental health problems and tobacco and cannabis use separately. In particular, tobacco use is associated with internalizing disorders, such as anxiety, depression, and stress,^8,9,10^ while cannabis use is closely linked with psychosis^11,12,13,14,15^ and externalizing behaviors, such as impulsivity and attention-deficit/hyperactivity disorders among youth.^16,17^ Concurrent use of tobacco and cannabis may be more concerning because it may lead to greater dependence on both substances.^15^ Several studies on adult populations have shown that mental health problems are more likely associated with tobacco and cannabis use among people who use both tobacco and cannabis compared with those who use tobacco or cannabis exclusively.^18,19,20,21^ Few studies have investigated potential additive negative effects of concurrent use on mental health, especially among youths whose brains are still developing.

Given the proliferation of tobacco and cannabis products in the past decade and an ongoing mental health crisis in youths,^6^ understanding how different patterns of tobacco and cannabis use, especially concurrent use, may be associated with mental health problems is important to inform public health efforts and improve youth well-being. Thus, we examined the prevalence of tobacco and cannabis use patterns and their associations with internalizing and externalizing mental health problems using the most recent youth survey data from the Population Assessment of Tobacco and Health (PATH) Study. Additionally, we compared the effect sizes of the association of concurrent tobacco and cannabis use and single-substance use (ie, tobacco-only and cannabis-only) with mental health problems. We hypothesized that concurrent use would be associated with the greatest odds of higher levels of mental health problems among youths.

## Methods

### Data Source and Participants

The PATH Study is an ongoing, nationally representative, longitudinal cohort study of adults and youths (aged ≥12 years). Baseline data (wave 1) were collected from January 2013 to December 2014, with 45 971 adults and youths. In addition, 7207 “shadow youths” (aged 9-11 years) were also sampled at wave 1 (for interviews when they turn 12 years of age in following waves), making up a total of 53 178 participants who constituted the wave 1 cohort. At wave 4, a probability sample of 14 098 adults, youths, and shadow youths aged 10 to 11 years was selected from the civilian, noninstitutionalized population at the time of wave 4. This sample was recruited from residential addresses not selected for wave 1 in the same sampled primary sampling units and segments using similar within-household sampling procedures. The replenishment sample at wave 4 together with wave 1 participants, 52 731 participants in total, formed the wave 4 cohort. Detailed information about the PATH Study’s data collection, study design, and methodology have been published elsewhere.^22,23^

The present study used publicly available wave 6 data (collected in person or via telephone from March to November 2021) that include a total of 5652 youths (aged 14-17 years). Public use data files and details on survey interview procedures, questionnaires, sampling, and weighting information are available online.^24^ The PATH Study was conducted by Westat and approved by the Westat Institutional Review Board, which waived the need for informed consent owing to the use of publicly available data. This study followed the Strengthening the Reporting of Observational Studies in Epidemiology (STROBE) reporting guideline.

### Measures

#### Outcome Variables: Internalizing and Externalizing Mental Health

Mental health problems (internalizing and externalizing) were measured using a modified version of the Global Appraisal of Individual Needs–Short Screener (GAIN-SS). The GAIN-SS is a validated, efficient, and reliable screening tool to identify people who are more likely to have mental health diagnoses and need clinical services.^25^ The internalizing subscale measures internalizing problems that cause internal psychological distress, such as anxiety and depression. The externalizing subscale measures externalizing problems that can cause interpersonal conflict in the external environment, such as impulsivity, hyperactivity, delinquency, and aggressive behaviors.^26^ The PATH Study questionnaires measured internalizing mental health problems using 4 of 5 internalizing items from the GAIN-SS, excluding the item asking about suicidal ideation, and measured externalizing mental health problems using all 5 GAIN-SS externalizing items plus 2 hyperactivity items from the full GAIN Behavioral Complexity Scale.^27^ Each item was scored 1 point if the measured symptom happened within the past year, creating a score range of 0 to 4 for internalizing problems and 0 to 7 for externalizing problems. We followed validated cut points to categorize internalizing and externalizing mental health problems into 3 levels: low (0-1 symptoms), moderate (2-3 symptoms), and high (≥4 symptoms).^25^ Those who report a low level of internalizing or externalizing problems are unlikely to need services, while a moderate level indicates a possible diagnosis and likelihood of benefit from a brief intervention, and a high level indicates a high probability of a diagnosis and need for more formal assessment and intervention.^25^ The measures of internalizing and externalizing mental health problems have previously been used with the PATH Study data.^9,28,29,30^

#### Independent Variable: Patterns of Tobacco and Cannabis Use

Tobacco and cannabis use patterns were derived from the past 30-day use of any tobacco products (yes or no) and past 30-day use of any cannabis products (yes or no) by categorizing 4 mutually exclusive groups, including nonuse (use of neither product), tobacco-only use, cannabis-only use, and concurrent use (use of both substances). Past 30-day use of tobacco was defined as self-reported use of any of the following products at least 1 day in the past 30 days: cigarette, traditional cigar, cigarillo, filtered cigar, pipe, hookah, bidi or kretek, e-cigarette, smokeless tobacco, snus, dissolvable tobacco, or heated tobacco. Past 30-day use of cannabis was defined as at least 1 day of vaping cannabis or marijuana liquids or oils, smoking dried herb or flower (in a joint, pipe, hookah, bong, cigar, cigarillo, and filtered cigar), or using cannabis or marijuana in some other way. In addition, we included those who reported using blunts in the past 30 days in the concurrent use category, as blunts typically consist of both cannabis and tobacco leaf.^31,32,33^

#### Covariates

Demographic variables included age (14 vs 15-17 years old), sex assigned at birth (male vs female), race and ethnicity, and parental highest educational level (less than high school, high school graduate or equivalent, some college without a degree or with an associate degree, bachelor’s degree, and advanced degree). Race and ethnicity were self-reported and were categorized as Hispanic, non-Hispanic Black, non-Hispanic White, and non-Hispanic Other (including American Indian or Alaska Native, Asian, Native Hawaiian or Other Pacific Islander, and more than 1 race); these data were included to account for racial and ethnic disparities associated with tobacco and cannabis use. Use of other substances included past 30-day use of alcohol and past 12-month use of cocaine or crack, stimulants, heroin, inhalants, solvents, or hallucinogens or misuse of any prescription drugs.

### Statistical Analysis

Data were analyzed from November 15, 2023, to April 20, 2024. All analyses were conducted using Stata, version 17 (StataCorp LLC). The PATH Study added a replenishment sample of youths at wave 4 (December 2016 to January 2018) to increase sample size and maintain national representation, forming a new wave 4 cohort.^23^ Thus, this analysis used wave 6 single-wave weights to obtain statistically valid estimates for cross-sectional analyses generalizable to the wave 4 cohort sample as per data user guidelines.^34^

Of the 5652 participants, the analytical sample included 5585 with weighted data, excluding 67 with missing weight information. Descriptive statistics estimated weighted prevalence and 95% CIs of tobacco and cannabis use patterns, as well as mental health problems. We used ordinal logistic regressions to examine the association between the 4 exclusive patterns of tobacco and cannabis use and each of the two 3-level outcomes (ie, internalizing and externalizing mental health problems). We confirmed the tenability of the proportional odds assumption for all models through Brant and Wolfe-Gould testing.^35^ For interpretation, we also switched the reference group of the independent variable from nonuse to tobacco use only and to cannabis use only to compare the effect sizes of the association across the tobacco and cannabis use patterns. All models adjusted for demographic covariates and the use of alcohol and other substances. All variances were estimated using the balanced repeated replication method,^36^ with Fay adjustment set to 0.3 to increase estimate stability^37^ as recommended in data user guidelines. All hypothesis tests were 2 sided, and *P* < .05 was considered statistically significant.

The proportions of missing data were low (<1%) for most variables, except race and ethnicity (5.6%) and internalizing problems (1.2%). There were no significant differences in the percentage of missing data across levels of mental health outcomes or tobacco and cannabis use patterns. We used the listwise deletion method to handle missingness and to incorporate replicated weights. As a robustness check, we repeated the analysis using multivariate imputation by chained equations with population weights and found no significant differences in the results with imputed datasets. Given that the multiple imputation method does not work well with the replicated weights, we report the models with complete numbers of cases.

## Results

Among the 5585 participants, 2635 (48.7%) were female and 2931 (51.3%) were male; 1271 (27.5%) were 14 years of age and 4314 (72.5) were aged 15 to 17 years. Most had at least 1 parent who attended some college or had a higher education (75.2%). In terms of race and ethnicity, 1606 (25.7%) were Hispanic, 626 (12.7%) were non-Hispanic Black, 2481 (50.5%) were non-Hispanic White, and 555 (11.0%) were non-Hispanic other. Regarding tobacco and cannabis use patterns, 3.9% of participants (95% CI, 3.2%-4.6%) reported tobacco use only, 2.5% (95% CI, 2.1%-2.9%) reported cannabis use only, and 3.4% (95% CI, 2.9%-4.0%) reported concurrent use of both tobacco and cannabis in the past 30 days. In addition, 9.1% (95% CI, 8.3%-9.9%) reported drinking alcohol in the past 30 days, and 4.9% (95% CI, 4.2%-5.6%) reported using other substances in the past 12 months. Substantial proportions of the sample reported moderate (28.4% [95% CI, 27.1%-29.7%]) or high (24.6% [95% CI, 23.3%-26.0%]) levels of internalizing problems, and proportions of moderate and high levels of externalizing problems were 30.7% (95% CI, 29.1%-32.3%) and 32.5% (95% CI, 31.1%-34.0%), respectively. Participant characteristics are provided in Table 1.

**Table 1.** Characteristics of US Youths Aged 14 to 17 Years^a^

Characteristic	Unweighted No. of participants (N = 5585)^a^	Weighted % (95% CI)
Demographic		
Sex assigned at birth		
Male	2931	51.3 (50.9-51.6)
Female	2635	48.7 (48.4-49.1)
Age, y		
14	1271	27.5 (26.5-28.5)
15-17	4314	72.5 (71.5-73.5)
Race and ethnicity		
Hispanic	1606	25.7 (25.2-26.3)
Non-Hispanic Black	626	12.7 (12.2-13.2)
Non-Hispanic White	2481	50.5 (49.8-51.2)
Non-Hispanic other^b^	555	11.0 (10.4-11.7)
Highest parental educational level		
Less than high school	491	7.8 (7.0-8.7)
High school graduate or equivalent	1008	17.0 (15.8-18.2)
Some college (no degree) or associate degree	1538	27.5 (25.9-29.3)
Bachelor’s degree	1221	23.1 (21.7-24.6)
Advanced degree	1289	24.6 (23.1-26.1)
Substance use		
Patterns of past 30-d tobacco and cannabis use		
Concurrent use	206	3.4 (2.9-4.0)
Tobacco only^c^	197	3.9 (3.2-4.6)
Cannabis only^d^	140	2.5 (2.1-2.9)
Nonuse	4997	90.2 (89.2 - 91.1)
Past 30-d alcohol use	500	9.1 (8.3-9.9)
Past 12-mo other substance use^e^	255	4.9 (4.2-5.6)
Mental health		
Internalizing mental health problem level		
Low (0-1 symptoms)	2653	47.0 (45.7-48.2)
Moderate (2-3 symptoms)	1555	28.4 (27.1-29.7)
High (≥4 symptoms)	1342	24.6 (23.3-26.0)
Externalizing mental health problem level		
Low (0-1 symptoms)	2078	36.8 (35.4-38.2)
Moderate (2-3 symptoms)	1697	30.7 (29.1-32.3)
High (≥4 symptoms)	1741	32.5 (31.1-34.0)

^a^ Data are from the Population Assessment of Tobacco and Health Study wave 6 in 2021. Owing to missing data, unweighted numbers may not total 5585.

^b^ Includes American Indian or Alaska Native, Asian, Native Hawaiian or Other Pacific Islander, and more than 1 race.

^c^ Includes cigarette, traditional cigar, cigarillo, filtered cigar, pipe, hookah, bidi or kretek, e-cigarette, smokeless tobacco, snus, dissolvable tobacco, or heated tobacco.

^d^ Includes vaping cannabis or marijuana liquids or oils, smoking dried herb or flower (in a joint, pipe, hookah, bong, cigar, cigarillo, or filtered cigar), or using cannabis or marijuana in some other ways.

^e^ Includes misuse of prescription drugs (ie, methylphenidate hydrochloride [Ritalin] or amphetamine [Adderall], painkillers, sedatives, or tranquilizers), cocaine or crack, stimulants, heroin, inhalants or solvents, and hallucinogens.

### Prevalence of Mental Health Problems Across Patterns of Tobacco and Cannabis Use

Overall, the proportions of respondents reporting a high level of mental health problems were higher among the tobacco and/or cannabis use groups compared with the nonuse group (Table 2). Particularly, 47.4% (95% CI, 39.2%-55.9%) of youths in the concurrent use group had a high level of internalizing problems, followed by 44.8% (95% CI, 35.7%-54.1%) of those in the cannabis-only use group, 41.4% (95% CI, 33.7%-49.5%) of those in the tobacco-only use group, and 22.4% (95% CI, 21.1%-23.8%) of those in the nonuse group. In addition, more than 6 of 10 (61.6% [95% CI, 54.1%-68.7%]) of the youths concurrently using tobacco and cannabis, about half of the youths using cannabis only (48.5% [95% CI, 39.1%-57.9%]), and 46.3% (95% CI, 38.3%-54.5%) of the youths using tobacco only had a high level of externalizing problems compared with 30.4% (95% CI, 28.9%-31.9%) of the youths who did not use tobacco or cannabis.

**Table 2.** Prevalence of Mental Health Problems by Patterns of Past 30-Day Tobacco and Cannabis Use Among US Youths Aged 14 to 17 Years^a^

Mental health problem level	Use group, weighted % (95% CI)			
	Concurrent use	Tobacco use only^b^	Cannabis use only^c^	Nonuse
**Internalizing (n = 5510)**				
None or low	26.6 (20.8-33.3)	31.3 (24.5-39.0)	26.8 (19.3-36.0)	49.0 (47.7-50.4)
Moderate	26.0 (18.6-35.0)	27.3 (21.2-34.3)	28.4 (21.8-36.1)	28.6 (27.3-29.9)
High	47.4 (39.2-55.9)	41.4 (33.7-49.5)	44.8 (35.7-54.1)	22.4 (21.1-23.8)
**Externalizing (n = 5478)**				
None or low	14.4 (9.8-20.6)	24.9 (18.5-32.7)	24.7 (17.1-34.3)	38.4 (36.8-40.0)
Moderate	24.0 (19.0-29.8)	28.8 (21.2-37.9)	26.8 (20.0-35.0)	31.2 (29.6-32.9)
High	61.6 (54.1-68.7)	46.3 (38.3-54.5)	48.5 (39.1-57.9)	30.4 (28.9-31.9)

^a^ Data are from the Population Assessment of Tobacco and Health Study wave 6 in 2021. None or low level of mental health problem indicates 0 to 1 symptoms; moderate, 2 to 3 symptoms; and high, 4 or more symptoms.

^b^ Includes cigarette, traditional cigar, cigarillo, filtered cigar, pipe, hookah, bidi or kretek, e-cigarette, smokeless tobacco, snus, dissolvable tobacco, or heated tobacco.

^c^ Includes vaping cannabis or marijuana liquids or oils, smoking dried herb or flower (in a joint, pipe, hookah, bong, cigar, cigarillo, or filtered cigar), or using cannabis or marijuana in some other ways.

### Association Between Patterns of Tobacco and Cannabis Use and Mental Health Outcomes

The associations between patterns of tobacco and cannabis use and mental health problems are shown in Table 3. After adjusting for covariates in the ordinal logistic regression model, the odds of having a higher level of internalizing mental health problems among youths who reported concurrent use were 2.32 (95% CI, 1.64-3.29) times the odds of those who used neither substance. Youths who used tobacco only (adjusted odds ratio [AOR], 1.95 [95% CI, 1.39-2.74]) or cannabis only (AOR, 2.32 [95% CI, 1.56-3.45]) also had higher odds of having higher levels of internalizing problems compared with their peers who used neither substance. There were no statistically significant differences between concurrent use and single-substance use groups regarding the odds of having internalizing problems.

**Table 3.** Adjusted Associations Between Past 30-Day Tobacco and Cannabis Use Patterns and Mental Health Problems Among US Youths Aged 14 to 17 Years^a^

Comparison	Mental health problems			
	Internalizing (n = 5130)		Externalizing (n = 5101)	
	AOR (95% CI)	*P* value	AOR (95% CI)	*P* value
Comparison with nonuse				
Concurrent use	2.32 (1.64-3.29)	<.001	3.10 (2.14-4.49)	<.001
Tobacco only use^b^	1.95 (1.39-2.74)	<.001	1.69 (1.26-2.27)	.001
Cannabis only use^c^	2.32 (1.56-3.45)	<.001	1.68 (1.12-2.51)	.01
Comparison with tobacco-only use				
Concurrent use	1.19 (0.77-1.83)	.42	1.83 (1.15-2.91)	.01
Cannabis-only use	1.19 (0.71-1.99)	.49	0.99 (0.62-1.59)	.97
Comparison with cannabis-only use				
Concurrent use	1.00 (0.61-1.64)	.99	1.85 (1.11-3.06)	.01
Age 15-17 vs 14 y	1.07 (0.93-1.22)	.33	0.83 (0.73-0.96)	.01
Female vs male	3.07 (2.73-3.45)	<.001	1.47 (1.31-1.66)	<.001
Race				
Hispanic	0.85 (0.73-0.98)	.03	0.81 (0.69-0.96)	.01
Non-Hispanic Black	0.70 (0.57-0.87)	.001	0.77 (0.63-0.94)	.01
Non-Hispanic White	1 [Reference]		1 [Reference]	
Non-Hispanic other^d^	0.97 (0.78-1.21)	.77	0.86 (0.70-1.04)	.12
Highest parental educational level				
Less than high school	1 [Reference]		1 [Reference]	
High school graduate or equivalent	1.12 (0.86-1.46)	.41	1.30 (1.01-1.67)	.04
Some college (no degree) or associate degree	1.48 (1.12-1.96)	.006	1.85 (1.38-2.48)	<.001
Bachelor’s degree	1.22 (0.92-1.61)	.16	1.76 (1.31-2.37)	<.001
Advanced degree	0.98 (0.72-1.35)	.92	1.92 (1.40-2.63)	<.001
Past 30-d alcohol use, yes vs no	1.44 (1.15-1.81)	.002	1.60 (1.29-1.97)	<.001
Past 12-mo other substance use, yes vs no	2.29 (1.70-3.08)	<.001	2.11 (1.61-2.77)	<.001

Abbreviation: AOR, adjusted odds ratio.

^a^ Data are from the Population Assessment of Tobacco and Health Study wave 6 in 2021. Adjusted ORs were estimated from the ordinal logistic regression models for each outcome variable (internalizing and externalizing mental health problems). The outcome variables were coded as 1 for low level (0-1 symptoms), 2 for moderate level (2-3 symptoms), and 3 for high level (≥4 symptoms). All *P* values for the proportional odds assumption tests (ie, Brant, Wolfe-Gould, and likelihood ratio) were larger than .05, indicating the proportional odds assumptions are reasonable for both models with internalizing and externalizing outcomes. A total of 455 observations for the model with internalizing outcome and 484 observations for the model with externalizing outcome were excluded due to missing information on outcome variables or covariates.

^b^ Includes cigarette, traditional cigar, cigarillo, filtered cigar, pipe, hookah, bidi or kretek, e-cigarette, smokeless tobacco, snus, dissolvable tobacco, or heated tobacco.

^c^ Includes vaping cannabis or marijuana liquids or oils, smoking dried herb or flower (in a joint, pipe, hookah, bong, cigar, cigarillo, or filtered cigar), or using cannabis or marijuana in some other ways.

^d^ Includes American Indian or Alaska Native, Asian, Native Hawaiian or Other Pacific Islander, and more than 1 race.

Regarding the externalizing mental health problems, the adjusted odds of having higher levels of problems among youths using both tobacco and cannabis (AOR, 3.10 [95% CI, 2.14-4.49]), using tobacco only (AOR, 1.69 [95% CI, 1.26-2.27]), and using cannabis only (AOR, 1.68 [95% CI, 1.12-2.51]) were higher compared with youths who used neither substance. Notably, concurrent use was associated with almost twice the odds of reporting higher levels of externalizing mental health problems compared with tobacco-only use (AOR, 1.83 [95% CI, 1.15-2.91]) and cannabis-only use (AOR, 1.85 [95% CI, 1.11-3.06]).

Covariates associated with higher odds of having internalizing and externalizing mental health problems were being female (vs male), reporting past 30-day alcohol use, and reporting past 12-month other substance use. In addition, youths aged 14 years (vs 15-17 years) and those who reported having a parent who completed high school or a higher educational level (vs less than high school) had greater odds of having higher levels of externalizing mental health problems. In contrast, non-Hispanic Black and Hispanic youths were less likely to report higher levels of internalizing and externalizing mental health problems compared with their White peers.

## Discussion

This cross-sectional study extends the literature by evaluating the prevalence of tobacco and cannabis use patterns in a nationally representative sample of US youths aged 14 to 17 years and identifying the differential associations between substance use patterns and mental health problems. We found that concurrent use of tobacco and cannabis was as common as tobacco-only use and even more common than cannabis-only use among youths. Additionally, mental health problems were common among youths who reported using tobacco and/or cannabis, with those who were using both substances having the highest proportion of reporting high levels of internalizing and externalizing problems. Furthermore, as hypothesized, concurrent use of tobacco and cannabis was linked to greater odds of having higher levels of externalizing problems compared with the exclusive use of either substance. However, this association did not hold for internalizing problems.

The prevalence of concurrent use of tobacco and cannabis (3.4%) in this study seemed to be lower compared with 5.4% among youths in 2014 from the NSDUH data^3^ or 12.4% among middle and high school students in California in 2019,^38^ which is consistent with a decreasing trend in tobacco and cannabis use observed among youths in recent years.^1,39,40,41^ The lower prevalence of tobacco, cannabis use, and concurrent use reported in 2021 could also be a temporary drop due to the impact of the COVID-19 pandemic (eg, effects of lockdown or disrupted supply chains).^42,43^ Studies have shown that tobacco and cannabis can be a gateway or reverse gateway for each other,^44^ and as a consequence, a rise in the use of one substance could lead to an increase in the concurrent use of both substances. In addition, as the legalization of cannabis has been expanding in the US,^45^ more youths may have access to cannabis, which may increase both cannabis use and concurrent use of cannabis and tobacco. Thus, it is important to continue to monitor the concurrent use of tobacco and cannabis among youths.

We found that mental health problems were commonly comorbid with tobacco and cannabis use in youth, especially among those who use both substances, as well as other substance use. These data complement prior research documenting poor mental health among youths during the COVID-19 pandemic.^46^ Taken together, these findings highlight a crucial need for comprehensive prevention and treatment interventions integrating concurrent cessation of tobacco and cannabis use with mental health support to tackle this comorbidity among youths. According to data from the NSDUH in 2022, among 922 000 youths aged 12 to 17 years with co-occurring mental health issues and substance use disorders in the past year, only 20.8% of them received treatment for this comorbidity, 50.8% received treatment for only 1 type of issue, and 28.4% received neither type of treatment.^2^ These data suggest that youths with comorbidity of substance use disorder and mental health issues have not been receiving sufficient attention and support from related stakeholders (eg, family, physicians providing mental health treatment, or services for cessation of tobacco and cannabis use). Tobacco and cannabis cessation programs should include screening and should provide mental health support. At the same time, physicians who provide mental health treatments for youths should prioritize screening for tobacco and cannabis use and offer integrated cessation support and services to those in treatment for mental health.

We found that concurrent use of tobacco and cannabis was associated with greater odds of having higher levels of externalizing problems, which supports the hypothesis of additive adverse effects of concurrent use on externalizing mental health. Previous studies have indicated a positive association between cannabis use and externalizing problems among youth.^11,12,13,14,15,16,17^ In addition, animal^47^ and human^48^ studies suggest that nicotine may interact with Δ9-tetrahydrocannabinol synergistically to increase the subjective and physiological effects of cannabis, which may suggest a mechanism for the observed higher effects of concurrent use compared with exclusive single substance use on externalizing problems in our study. On the other hand, we did not observe greater odds of concurrent use (vs exclusive single substance use) associated with internalizing problems. Our study used cross-sectional data, so future longitudinal studies with biomarkers are needed to confirm the results and further explore the mechanisms of the potential additive effects of concurrent use on mental health, particularly for externalizing problems.

### Limitations

Our study has some limitations. Because of the cross-sectional design of the study, it was not possible to determine causal relationships between tobacco and cannabis use and levels of internalizing and externalizing problems. In addition, despite having a national sample of youths, participants in wave 6 of the PATH Study were not perfectly representative of youths sampled at wave 4, as those who became 18 years of age at wave 5 or 6 moved to the adult survey. The concept of concurrent use in our study was defined as the past 30-day use of any tobacco and cannabis, while we were unable to examine other concurrent use patterns (ie, same month and different day, same day and different occasion, same occasion and sequential, and same occasion and simultaneous)^49^ due to the lack of detailed measures. We were also unable to examine the dose-response effects between the frequency and intensity of use of tobacco and cannabis and mental health due to the lack of consistency and availability of measures of use frequency across a variety of tobacco and cannabis products. While some studies found the COVID-19 pandemic was associated with a decrease in tobacco and cannabis use, others found youths experienced increased mental health problems during the pandemic due to factors such as isolation, loneliness, lack of physical exercise, and family stress.^50^ This study lacked measures to quantify and control for the impact of the COVID-19 pandemic on the findings. Finally, self-reported data are subject to recall and social desirability bias.

## Conclusions

The findings of this cross-sectional study suggest that there is a high prevalence of internalizing and externalizing problems among US youths who used tobacco and cannabis, especially those who were using both substances. Our findings also suggest an association of the concurrent use of tobacco and cannabis with externalizing problems. These findings highlight a critical need for public health interventions addressing youth mental health and clinicians providing mental health screening and treatment to also address the comorbidity of tobacco and cannabis use. Programs for cessation of tobacco and cannabis use should also provide mental health support. Integrated treatment of mental health and tobacco, cannabis, and other substance use may be a more efficient use of resources, and continued surveillance is needed to monitor the impact of cannabis legalization on youth use, use concurrent with tobacco, and mental health.

## Notes

Supplement.
Data Sharing Statement
