# Supplementary material for: Concurrent Use of Tobacco and Cannabis and Internalizing and Externalizing Problems in US Youths
Source: JAMA Netw Open. 2024 Jul 3;7(7):e2419976. doi: 10.1001/jamanetworkopen.2024.19976 (PMC11222996; doi:10.1001/jamanetworkopen.2024.19976)
Supplement: Supplement. — Data Sharing Statement [file jamanetwopen-e2419976-s001.pdf]

Data Sharing Statement

Do. Concurrent Use of Tobacco and Cannabis and Internalizing and Externalizing Problems in US Youths. *JAMA Netw Open*. Published July 03, 2024.
doi:10.1001/jamanetworkopen.2024.19976

Data
Data available: Yes
Data types: Deidentified participant data
How to access data: https://www.icpsr.umich.edu/web/NAHDAP/studies/36498/datadocumentation#
When available: With publication

Supporting Documents
Document types: None

Additional Information
Who can access the data: anyone requesting the data
Types of analyses: for any purpose
Mechanisms of data availability: with investigator support
Any additional restrictions: none
